# Aromatase Inhibition Attenuates Desflurane-Induced Preconditioning against Acute Myocardial Infarction in Male Mouse Heart *In Vivo*

**DOI:** 10.1371/journal.pone.0042032

**Published:** 2012-08-02

**Authors:** Virginija Jazbutyte, Jan Stumpner, Andreas Redel, Johan M. Lorenzen, Norbert Roewer, Thomas Thum, Franz Kehl

**Affiliations:** 1 Institute of Molecular and Translational Therapeutic Strategies (IMTTS), Hannover Medical School (MHH), Hannover, Germany; 2 Department of Anesthesia and Critical Care, University of Würzburg, Würzburg, Germany; 3 Department of Anesthesia, University of Regensburg, Regensburg, Germany; 4 Department of Anesthesiology and Critical Care, Klinikum Karlsruhe, Karslruhe, Germany; University of Colorado Denver, United States of America

## Abstract

The volatile anesthetic desflurane (DES) effectively reduces cardiac infarct size following experimental ischemia/reperfusion injury in the mouse heart. We hypothesized that endogenous estrogens play a role as mediators of desflurane-induced preconditioning against myocardial infarction. In this study, we tested the hypothesis that desflurane effects local estrogen synthesis by modulating enzyme aromatase expression and activity in the mouse heart. Aromatase metabolizes testosterone to 17β- estradiol (E2) and thereby significantly contributes to local estrogen synthesis. We tested aromatase effects in acute myocardial infarction model in male mice. The animals were randomized and subjected to four groups which were pre-treated with the selective aromatase inhibitor anastrozole (A group) and DES alone (DES group) or in combination (A+DES group) for 15 minutes prior to surgical intervention whereas the control group received 0.9% NaCl (CON group). All animals were subjected to 45 minutes ischemia following 180 minutes reperfusion. Anastrozole blocked DES induced preconditioning and increased infarct size compared to DES alone (37.94±15.5% vs. 17.1±3.62%) without affecting area at risk and systemic hemodynamic parameters following ischemia/reperfusion. Protein localization studies revealed that aromatase was abundant in the murine cardiovascular system with the highest expression levels in endothelial and smooth muscle cells. Desflurane application at pharmacological concentrations efficiently upregulated aromatase expression *in vivo* and *in vitro.* We conclude that desflurane efficiently regulates aromatase expression and activity which might lead to increased local estrogen synthesis and thus preserve cellular integrity and reduce cardiac damage in an acute myocardial infarction model.

## Introduction

Cardioprotective events following application of volatile anesthetics prior to ischemia/reperfusion are called anesthetic preconditioning [1]. The mechanisms involved in cardioprotection by volatile anesthetics, such as sevoflurane and desflurane, include increased nitric oxide (NO) synthesis, activation of mitochondrial large- conductance calcium- activated potassium channel, beta1- adrenergic pathway and generation of reactive oxygen species (ROS) [2]–[5]. Anesthetic preconditioning is similar to and shares many common signal transduction pathways with ischemic preconditioning which is characterized by brief repeated periods of vascular occlusion prior to prolonged ischemia/reperfusion which protects ischemic myocardium [6]. Despite that, the phenomenon of anesthetic preconditioning is not fully understood and little is known about the mediators of anesthetic preconditioning. The major female sex hormone 17β- estradiol (E2) was shown to be cardioprotective in ischemia/reperfusion injury model in ovariectomized rats and rabbits [7], [8]. Wang et al demonstrated that volatile anesthetic isoflurane - mediated cardioprotection was efficient in male but not in female rabbits. The authors concluded that smaller infarct size was dependent on female gender and application of isoflurane did not produce additional cardioprotection in female rabbits *in vivo* [9]. The major female sex hormone 17β- estradiol is synthesized from testosterone where the last step of synthesis is catalyzed by the enzyme aromatase (P450arom). 17β- estradiol is mainly produced in the female reproductive system and some other organs, such as adipose tissue, liver and adrenal glands. In males, estrogen synthesis takes place in the testes and some peripheral organs, such as adipose tissue, regions of the brain and bone [10]. Aromatase deficiency in both, females and males, were associated with undetectable estrogens and impaired fertility of female mice [11]. Additionally, aromatase disruption in mouse model (ArKO mice) resulted in increased adiposity, altered fatty acid metabolism and higher levels of testosterone, the features characteristic for metabolic syndrome [12]. Aromatase expression analysis in the cardiovascular system showed that aromatase mRNA was abundant in endothelial cells and also smooth muscle cells [13]–[15]. Functional analysis of aromatase deficiency in the cardiovascular system of female mice showed that ArKO mice had reduced diastolic and mean blood pressure together with increased blood pressure variability [16]. Aromatase inhibition in healthy young men caused significant decrease in serum estrogen levels and reduced vasodilation without altering other parameters, such as serum testosterone, lipoprotein levels and endothelium- independent vasodilation [17].

In the current study, we determined whether the volatile anesthetic desflurane modulates aromatase expression and/or activity in the heart and thus may contribute to cardioprotection in an acute ischemia/reperfusion model.

## Materials and Methods

### 1. Ethics Statement

Animal treatment and surgery were performed according to recommendations of the Guide for the Care and Use of Laboratory Animals published by the United States National Institute of Health. All experimental protocols were reviewed and approved by the Animal Care and Use Committee of the Government of Lower Franconia, Bavaria, Germany.

### 2. Animals and Surgery

**Figure 1:**
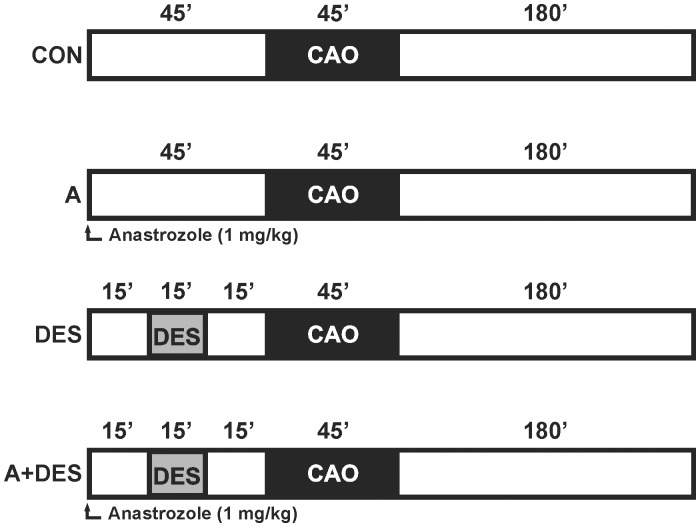
Schematic diagram illustrating the experimental protocol. CON = control; CAO = coronary artery occlusion; A = anastrozole (1 mg/kg BW); DES = desflurane (1 minimum alveolar concentration); A+DES = anastrozole and desflurane at concentrations indicated above. Control experiment (CON) comprises 45 minutes of coronary artery occlusion (CAO) and subsequent 180 minutes long reperfusion. Anastrozole in A group was administrated intravenously 45 minutes prior to CAO and subsequent 180 minutes reperfusion. In DES group, desflurane was applied at 1 MAC concentration for 15 minutes 30 minutes before CAD and subsequent reperfusion. In A+DES group, anastrozole was injected intravenously 15 minutes before desflurane application; CAO and reperfusion were performed as indicated above.

Male C57/BL6N mice (8–12 weeks) were purchased by Charles River Laboratories (Sulzfeld, Germany). Animals were housed under controlled conditions (22°C, 55%–65% humidity and artificial 12 hour light/dark regime) and had free access to standard laboratory chow and tap water. A total of forty (n = 6–10 animals/group) were randomly assigned to following experimental groups: control group (CON) which underwent ischemia/reperfusion surgery, A group which received the aromatase inhibitor Anastrozole (Arimidex®, AstraZeneca Pharmaceuticals Group, Alderley Park, UK) prior to ischemia/reperfusion, DES group received 1 minimum alveolar concentration (MAC) of Desflurane (Baxter Deutschland GmbH) prior to ischemia/reperfusion and DES+A group obtained Anastrozole and subsequent DES treatment prior to ischemia/reperfusion. The mice were anesthetized with sodium pentobarbital, intubated and mechanically ventilated. Anastrozole was dissolved in 0.9% NaCl and injected intravenously at a dosage of 1 mg/kg (max. vol. 100 µl). Mean arterial blood pressure and heart rate were measured via cannulation of the right carotid artery. Coronary artery occlusion (CAO) was performed by 45 minutes ligation of the left anterior descending coronary artery (LAD) using a 6-0 silk suture. Subsequently, the ligature was removed and the hearts were reperfused 180 minutes. A schematic diagram of the experimental protocol is shown in figure 1.

For aromatase gene expression regulation experiments, mice were anesthetized with sodium pentobarbital, intubated and mechanically ventilated. The mice were treated with desflurane (1 MAC) for 15 minutes, awoken from anesthesia and kept under standard conditions for 15 minutes (DES15 group) or 48 hours (DES48 group) as described at the beginning of this chapter. Afterwards, the hearts were collected, shock-frozen in liquid nitrogen and subjected to RNA isolation and western blotting assays.

### 3. Measurement of Myocardial Infarct Size

Area at risk (AAR) and infarct size (IS) were quantified using Evans Blue staining as described previously [18], [19]. After 45 minutes of ischemia and 180 minutes reperfusion, the LAD was reoccluded and 1 ml of Evans blue (Sigma- Aldrich, St. Louis, USA) was injected into the carotid artery. The hearts were explanted, cut into 1 mm thick transversal slices, incubated with 2% triphenyltetrazolium chloride (Sigma- Aldrich, St. Louis, USA), fixed overnight in 10% formalin, weighed, and digitally photographed. The photographs were then analyzed with AdobePhotoshop CS 8.0.1 software. Infarct size, area at risk and normal zone were quantified by an investigator blinded to the treatment protocol. The resulting fractions of infarct size, area at risk and normal zone of each slice were multiplied by the weight of that slice. Animals with an AAR<15% of the left ventricle were excluded from the study. Myocardial infarct size (IS) is expressed as a percentage of the left ventricular area at risk (AAR).

### 4. Systemic Hemodynamics

During experiments, heart rate (HR, beats/min), mean arterial pressure (MAP, mmHg) were recorded using a saline-filled PE-10 catheter connected to a pressure transducer (Combitrans, B. Braun, Melsungen, Germany) which was inserted into the right common carotid artery. The parameters were recorded at the end of the baseline period (BL), at the end of the intervention period (desflurane or corresponding time point), at the end of memory period (MEM, memory period), at the end of coronary artery occlusion ( = CAO) and 60, 120 and 180 min after the onset of reperfusion. The rate pressure product (RPP) which is an indicator of myocardial oxygen consumption was calculated using following formula: RPP = HRxMAP/1000.

**Figure 2:**
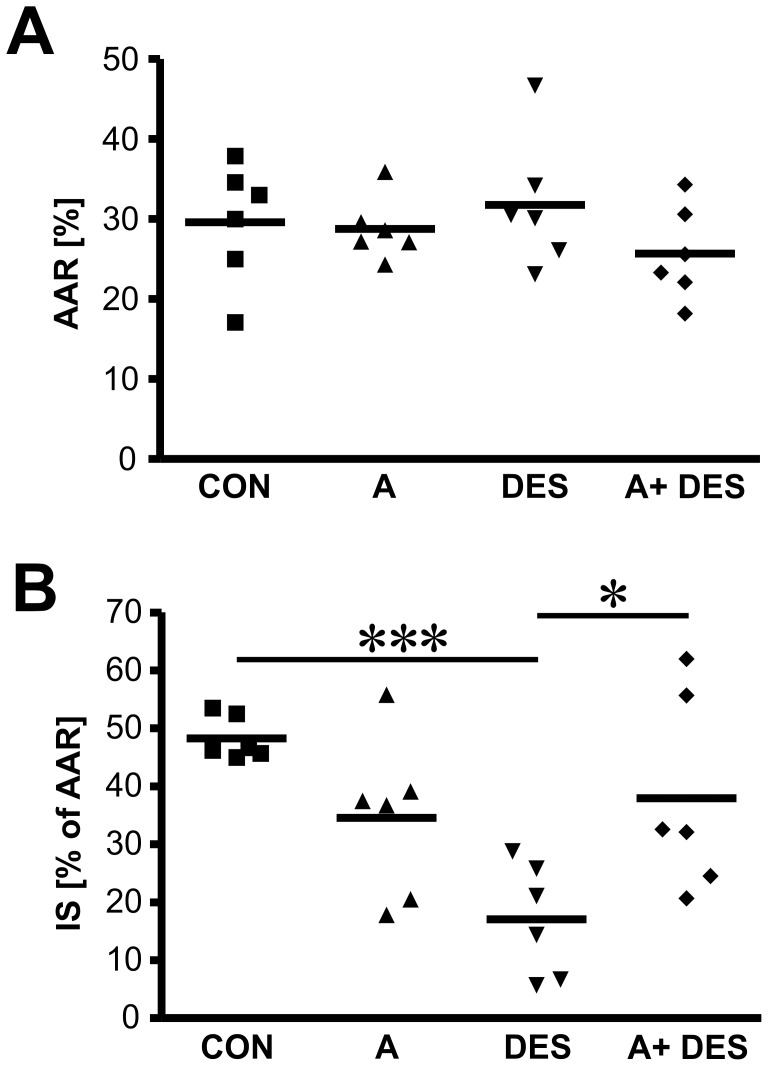
Summary charts of area at risk and infarct size measurements in male mice hearts. After hemodynamic measurements, the animals were perfused with triphenyltetrasolium (TPP) and Evans Blue to assess perfused myocardium, area at risk and infracted (necrotic) tissue. Myocardial infarct size (IS) was expressed as a percentage of area at risk (AAR). Values are mean percent± SEM, n = 6 animals/group. A *P* value <0.05 was considered as statistically significant.

### 5. RNA Isolation and Quantitative Real-time PCR

Total RNA was extracted from mouse heart using Trizol (Invitrogen, Carlsbad, USA) according to the manufacturer’s recommendations. The cDNA was generated using First- strand cDNA synthesis kit (Biorad, Munich, Germany). Aromatase gene Cyp19a1 and GAPDH expression analysis was performed using quantitative real- time PCR method. PCR master mix and primer mix were purchased from SABiosciences (Biomol, Hamburg, Germany), PCR reactions were carried out with a DNA Engine Opticon 2 System (MJ Research, Biozym, Oldendorf, Germany) and the data were analyzed using delta delta C(t) method (ΔΔC(t)).

**Table 1:** The results of area at risk and infarct size measurements in cardiac ischemia/reperfusion model.

Group	*n*	Area at risk [%]	Infarct size [% of AAR]
CON	6	29.6±2.8	48.3±1.4*
A	6	28.8±1.5	34.6±5.2 ^§^
DES	6	31.8±3.1	17.1±3.6* ^#^^§^
A+DES	6	25.7±2.2	37.9±15.5 ^#^

Data: Mean [%] ± SEM.

* , ^#^, ^§^- statistically significant differences, p<0.05.

CON =  control group; A =  Anastrozole group; DES =  desflurane group; A+DES =  anastrozole+ desflurane.

### 6. Cell Culture

Human umbilical cord endothelial cells (HUVEC) were extracted from umbilical cords using Trypsin-EDTA (Sigma-Aldrich, St. Louis, USA) digestion and cultivated in M199 medium (Gibson- Invitrogen, Carlsbad, USA) supplemented with 20% FCS, endothelial cell growth supplement (ECGF) (BD Biosciences, Bedford, USA), heparin (Liquemin®N, Hoffmann-La Roche AG, Grenzach-Wyhlen, Germany) and penicillin/streptomycin (Sigma- Aldrich, St. Louis, USA). Briefly, the umbilical cords were washed several times with PBS and endothelial cells were extracted following trypsin digestion. The cells were cultivated in Medium 199 with supplements and subjected for in *vitro* experiments. For in vitro experiments, HUVECs were treated with oxygen (1 liter/h, control group, CON) or oxygen: desflurane mixture containing 7.5 vol. % and comprising 1 mean alveolar concentration (MAC) of desflurane for 15 minutes in a closed chamber (DES group). Following 15 minutes treatment, cells were transferred to the incubator and further kept under standard conditions (5% CO2, 37°C). Cells were collected 15 minutes (DES 0.25), 24 hours (DES24) or 48 hours (DES48) later and subjected to further analysis. Cells from the native group were kept under standard conditions and collected together with control and desflurane-treated cells.

**Table 2:** Invasive hemodynamics results.

	BL 15′		DES 15′		MEM 15′		CAO 45′		Reperfusion					
HR (min^−1^)									60 min		120 min		180 min	
**CON**	447	±19	464	±25	463	±29	458	±21	450	±13	465	±18	460	±13
**A**	467	±21	466	±19	464	±18	448	±14	467	±14	477	±13	477	±30
**DES**	473	±17	417	±11	480	±19	432	±23	452	±13	447	±6	463	±15
**DES+A**	468	±19	448	±17	460	±21	495	±26	483	±18	489	±27	455	±19
**MAP(**mmHg)														
**CON**	71	±3	71	±3	66	±5	60	±1	67	±4	63	±4	66	±5
**A**	67	±6	61	±5	58	±4	50	±5	61	±7	58	±7	68	±6
**DES**	72	±2	73	±5	69	±3	61	±1	67	±1	67	±2	62	±2
**DES+A**	74	±3	80	±2	74	±7	66	±7	69	±8	58	±7	58	±8
**RPP** (HRxMAP/1000)														
**CON**	32	±3	33	±3	30	±3	27	±1	30	±2	30	±2	31	±3
**A**	30	±3	29	±3	27	±3	23	±3	29	±4	28	±3	33	±4
**DES**	34	±2	30	±2	33	±2	26	±1	31	±1	30	±1	29	±2
**DES+A**	36	±2	36	±2	34	±4	34	±4	34	±4	29	±4	27	±4

BL 15′, baseline; DES 15′, desflurane; MEM, memory; CAO, coronary artery occlusion; HR, heart rate; MAP, mean arterial pressure; RPP, rate pressure product.

Hemodynamic measurements were performed 15 minutes following equilibration period (baseline), at the end of desflurane application, at the end of memory phase (15 min.), at the end of 45 minutes of coronary artery occlusion and three times (at 60, 90 and 180 minutes) during reperfusion.

Primary rat aortic smooth muscle cells were cultivated in DMEM (Sigma- Aldrich, St. Louis, USA) containing 10% FCS. The cells were provided by Paula Anahi Arias- Loza from the Department of Cardiology, University Clinics Würzburg, Würzburg, Germany.

**Figure 3:**
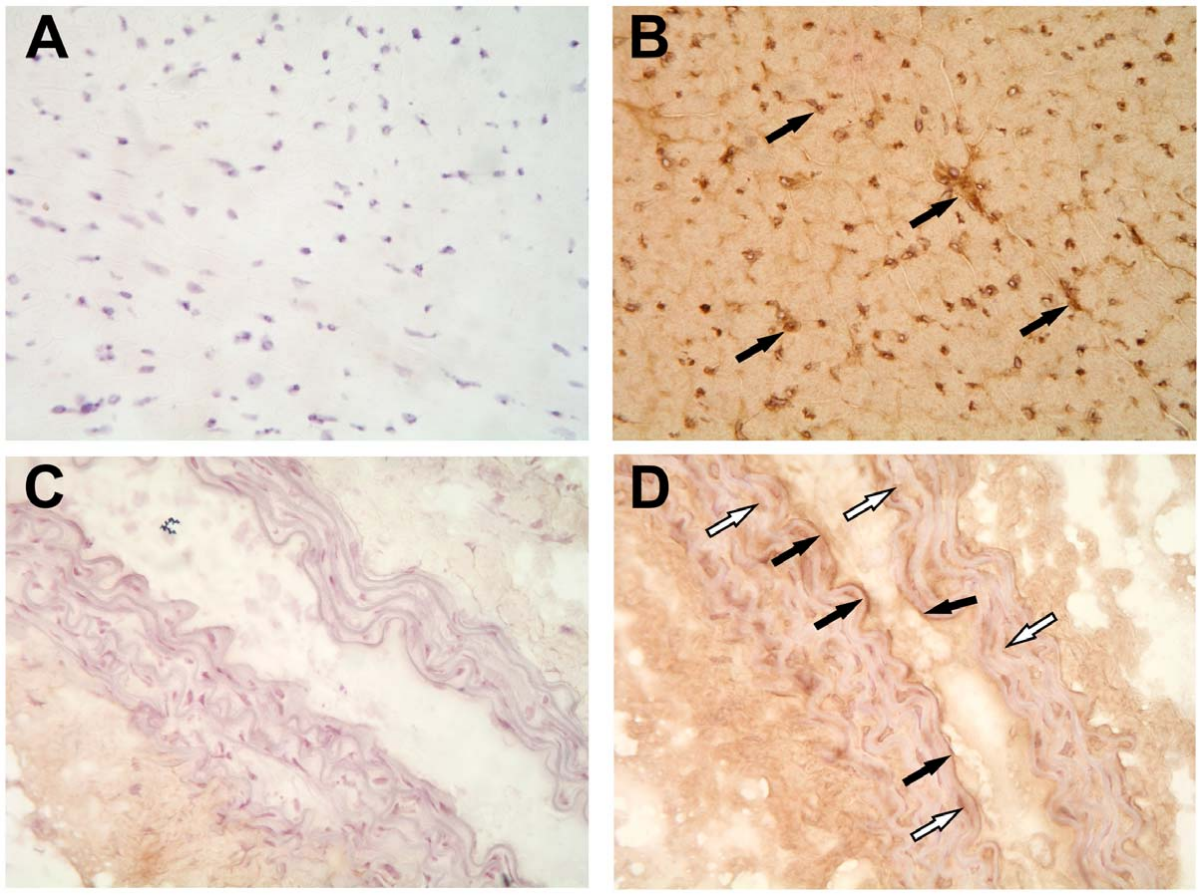
Aromatase immunostaining in mouse heart and aorta. Aromatase protein was abundant in mouse myocardium (B, solid arrows) and in the endothelium (solid arrows) as well as in media (white arrows) in the aorta (D). Aromatase signal was visualized by diaminobenzidine substrate (DAB) combined with hematoxylin/eosin staining. Primary antibody was omitted in negative control staining (A and C).

### 7. Immunostaining

Mouse heart tissue and the aorta were frozen in Tissue- Tec OCT compound (Sakura, Alphen an den Rijn, The Netherlands) and cut in 5 µm thick cryosections. The sections were fixated with 4% PFA, permeabilized with 0.1% Triton X-100 and subjected to immunostaining. Aromatase was detected using rabbit- anti- aromatase antibody (Biocat, Heidelberg, Germany), smooth muscle cells were visualized by Cy3- labelled mouse anti- smooth muscle actin antibody (Sigma- Aldrich, Hamburg, Germany), endothelial cells were stained using rat anti- CD31 (PECAM1) antibody (BD Biosciences/Pharmingen, Heidelberg, Germany) and cell nuclei were visualized using goat- anti- lamin A/C antibody (N-18) (Santa Cruz biotechnology, Santa Cruz, USA). Unspecific background was blocked using donkey serum diluted in PBS (Sigma- Aldrich, Hamburg Germany). Specific signals for immunofluorescence were visualized using AlexaFluor labelled donkey anti- rat, donkey- anti- rabbit and donkey- anti-goat antibodies purchased from Molecular Probes (now Invitrogen, Carlsbad, USA). In immunohistochemical staining, specific signal was detected using HRP- labelled secondary antibodies in combination with streptavidin- horse reddish peroxidase (HRP) and visualized by diaminobenzydine (DAB) system as described by manufacturers (Vector Laboratories, Burlingame, USA). Fluorescence imaging was performed using confocal laser scanning microscope MRC-1024 and the picture acquisition was performed using the Lasersharp2000 software (Biorad, Munich, Germany).

**Figure 4:**
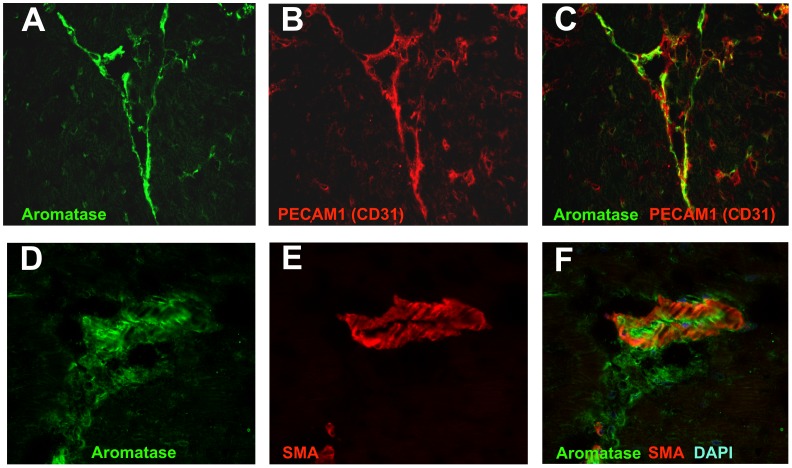
Aromatase co- localization with endothelial cells and smooth muscle cells in mouse heart studies using confocal microscopy. Aromatase was detected by immunostaining using specific antibody and visualized by AlexaFluor488- coupled secondary antibody (A and D). Endothelial cell specific marker PECAM1 (CD31) (B) and smooth muscle marker α-smooth muscle actin (SMA) (E) were detected using specific primary antibodies and visualized using AlexFluor594-coupled secondary antibodies (B and E, respectively). Aromatase and PECAM1 co- localization is shown in section C, whereas aromatase and α-smooth muscle actin co- expression in the heart is demonstrated in section F.

### 8. Western Blotting

Protein samples were prepared from untreated HUVEC cells (Native group), treated with oxygen: desflurane (DES group) or with pure oxygen (CON group). Briefly, cells were washed with ice- cold PBS and lysed for 15 minutes in cold RIPA buffer containing Complete protease inhibitor cocktail (Roche, Penzberg, Germany). Protein samples were electrophoretically separated and transferred overnight at 100 mA to nitrocellulose membrane. Next day, the membranes were blocked with 5% skimmed milk in PBS and aromatase protein expression was detected using rabbit- anti- aromatase antibody (Biocat, Heidelberg, Germany) at concentration 1 µg/ml in blocking solution. Aromatase signal was visualized using HRP - conjugated goat- anti- rabbit secondary antibody (dilution 1∶10000) (Cell signaling, Beverly, MA, USA) and Amersham ECL Plus western blotting detection reagent (GE Healthcare, Pittsburgh, PA, USA). We used GAPDH protein as an internal loading control, which was detected on the same membranes using mouse anti- GAPDH antibody (Chemicon, Billerica, MA,USA) at concentration 0.5 µg/ml, visualized using HRP- conjugated goat anti- mouse antibody (Cell signaling, Beverly, MA, USA) in combination with ECL Plus substrate. Aromatase and GAPDH signal was developed using X- ray film and signal intensity was quantified using Scion Image freeware (Scion corp). Signal intensity ratio between aromatase and GAPDH was statistically analyzed and the results are presented as mean values of four independent experiments.

**Figure 5:**
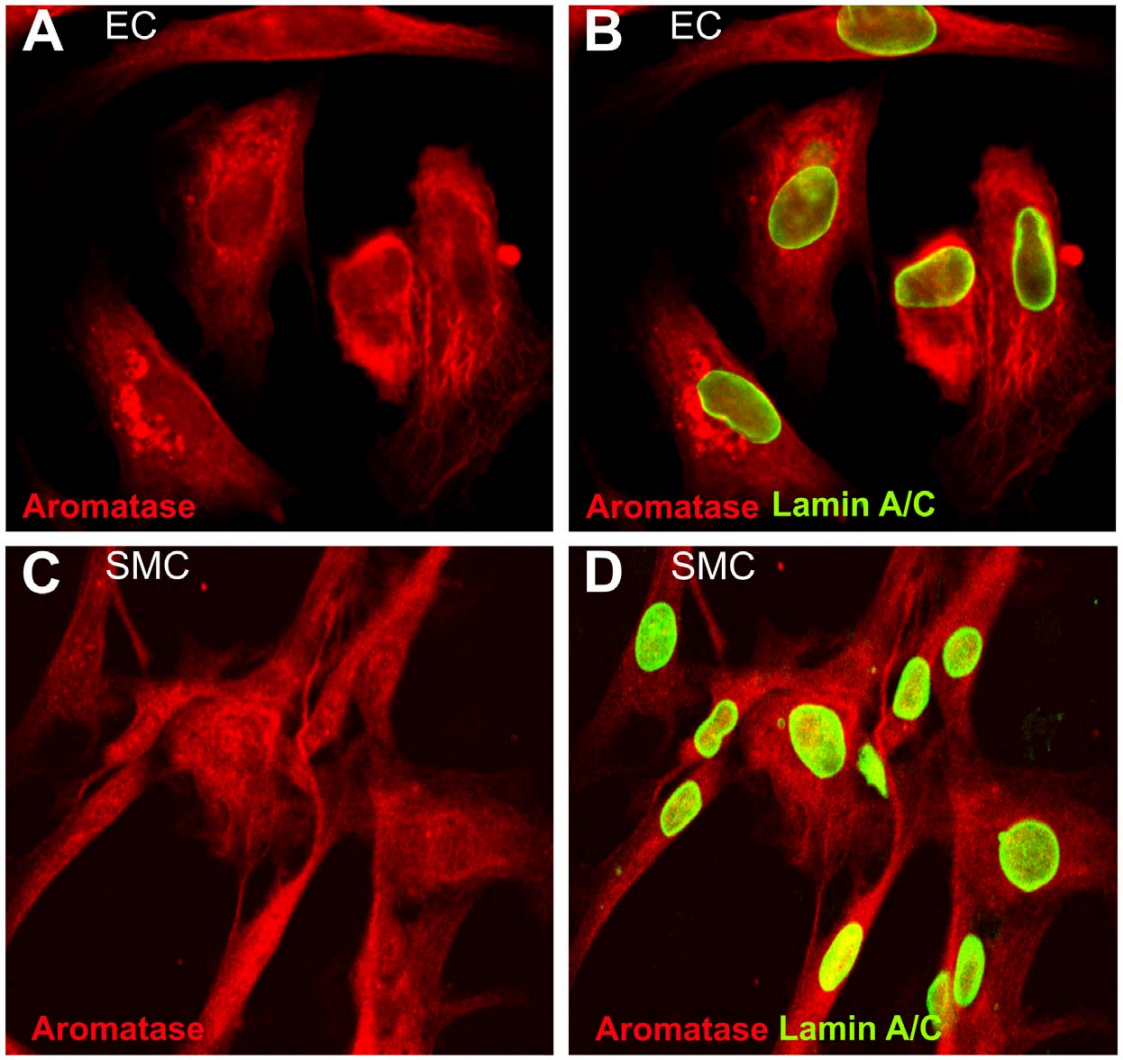
Aromatase expression in endothelial and smooth muscle cells. Aromatase cellular localisation pattern in endothelial (EC) and smooth muscle cells (SMC) was assessed by immunocytochemistry and visualized using confocal imaging technique. Aromatase was immunostained using specific primary antibody and visualized by addition of the secondary antibody conjugated with AlexaFluor594 fluorophore (red). Aromatase was abundant in the cytosolic fraction of endothelial (A) and smooth muscle cells (C). Cell nuclei were detected using lamin A/C antibody and visualized using AlexaFluor488- labelled secondary antibody (green). Merge images B and D show aromatase and lamin A/C co- immunostaining.

### 9. Statistics

Statistical analysis of data within and among groups was performed with one-way analysis of variance (ANOVA) followed by *post hoc* Student-Newman-Keuls method test using SigmaStat 2.0 software. A *P* value less than 0.05 was considered as statistically significant.

**Figure 6:**
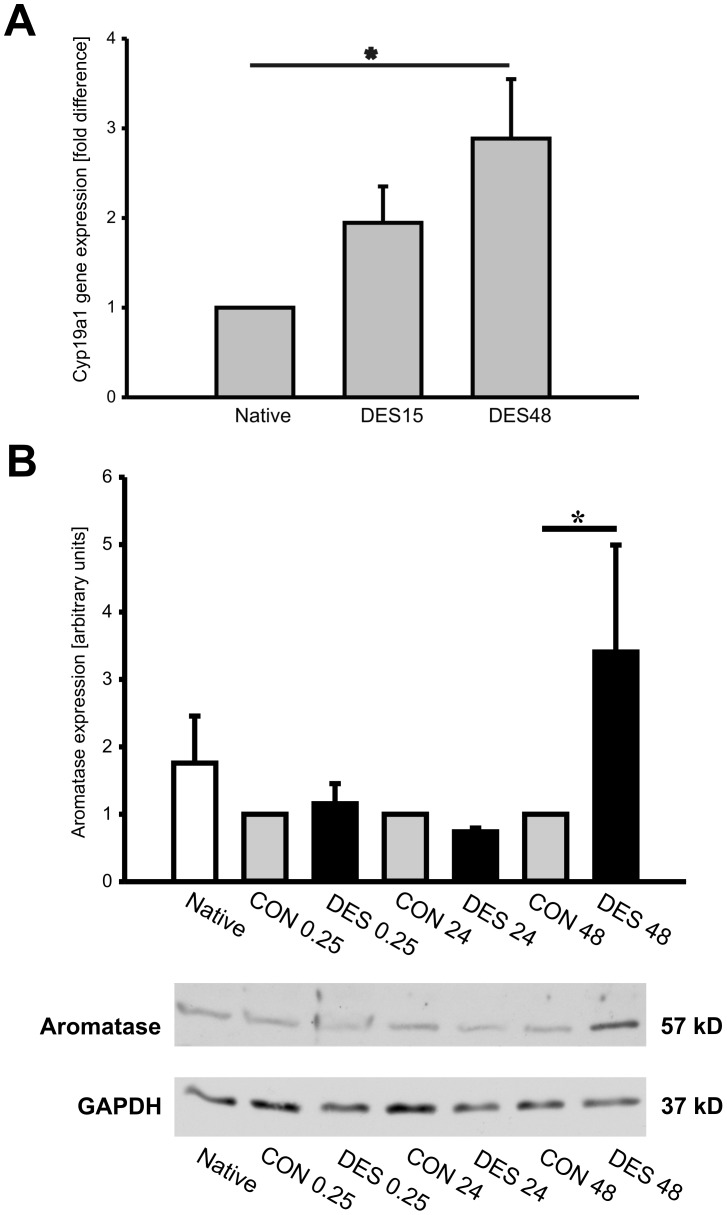
Aromatase upregulation by desflurane *in vivo* and *in vitro*. Male mice were treated with 1 MAC desflurane for 15 minutes, then were euthanized 15 minutes later (DES15) or 48 hours later (DES48). The hearts from untreated animals served as native control (Native). Total RNA was extracted from myocardial tissue and aromatase gene (Cyp19a1) expression was analyzed by quantitative real- time PCR (qRT-PCR), n = 5/group (A). Aromatase protein expression *in vitro* was studied by western blotting. Endothelial cells were treated with oxygen: desflurane (1 MAC) mixture for 15 minutes and collected 15 minutes later (DES0.25), 24 hours later (DES24) or 48 hours later (DES 48). Cells treated with oxygen alone for 15 minutes and collected 15 minutes later, 24 hours later and 48 hours later were assigned as controls (CON0.25, CON 24 and CON 48, respectively). The cells kept under standard conditions served as native control (B). The chart in panel B represents statistical analysis of aromatase signal quantification (n = 4). Aromatase signal in CON group was set to 1 (gray bars) and desflurane influence on aromatase protein expression was compared to CON group (solid bars). GAPDH protein served as internal loading control. Representative aromatase western blot is shown in lower part of panel B. The data are presented as mean± SEM and the *P* value <0.05 was considered as statistically significant.

## Results

### Area at Risk and Infarct Size Measurements

Area at risk and myocardial infarct size were measured to identify the levels of cardiac injury following ischemia/reperfusion. Area at risk values were comparable among all treatment groups (figure 2, panel A) whereas infarct size measurements demonstrated that desflurane alone (DES group) was superior in reduction of myocardial infarction size in male mice compared to the control group (CON) which underwent ischemia/reperfusion (17.1±3.62% vs. 48.3±1.4%, p<0.001) (figure 2, panel B). Interestingly, aromatase inhibition with anastrozole resulted in marginal decrease in infarct size (group A in figure 2B) but anastrozole application prior to desflurane treatment (group A+DES) significantly increased infarct size compared to DES alone (37.94±15.5% vs. 17.1±3.62%). Therefore, we conclude that DES- induced cardioprotection was at least partially mediated by endogenous locally synthesised estrogens in male mice in our experimental model. The data of area at risk and myocardial infarction size measurements are summarized in table 1.

### Systemic Hemodynamics

Anastrozole application did not influence cardiac function and there were no significant differences between the treatment groups. Hemodynamic data are summarized in table 2.

### Aromatase Localization in Male Mouse Heart

We performed aromatase localization studies to assess aromatase protein expression and its localization pattern in the cardiovascular system of male mice. Immunostaining results showed that aromatase was highly expressed in the myocardium and in the aorta (Fig. 3 B and D). Aromatase protein was abundant in cardiac vasculature and, to lesser extent, in cardiomyocytes (Fig. 3B). Aromatase was also detectable in intima and media in the aorta (Fig. 3D, solid arrows and white arrows, respectively). Using confocal imaging technique, we could confirm immunohistochemistry data and show that aromatase was expressed in the murine heart (Fig. 4 A and D) and co-localized with endothelial cells which were detected using endothelial cell marker PECAM1 (CD31) specific antibody (Fig. 4, B and C). Additionally, we observed partial aromatase co- localization with smooth muscle cells, which were visualized using smooth muscle actin- specific antibody (Fig. 4, E and F). Further cellular localization studies revealed strong aromatase expression in the cytosol of both, human endothelial (Figure 5, A and B) and aortic smooth muscle cells (Figure 5, C and D).

### Aromatase Gene Expression is Upregulated by Desflurane

To answer the question whether desflurane is able to regulate aromatase gene expression *in vivo*, we treated male mice with 1 MAC desflurane for 15 minutes and collected the hearts 15 minutes and 48 hours later. Aromatase gene *Cyp19a1* expression regulation by desflurane was assessed by quantitative real- time PCR. The data showed that aromatase mRNA expression was upregulated by desflurane as early as 15 minutes following desflurane application and significant increase in *Cyp19a1* expression levels was observed following 48 hours (figure 6A). Aromatase protein expression in the heart was comparable among the groups (data not shown). To confirm *in vivo* data, we treated human endothelial cells (HUVECs) with a mixture of desflurane and oxygen (1 MAC (7.5 vol%) and 92.5% oxygen) for 15 minutes, transferred the cells to the incubator with standard conditions (5% CO_2_, 37°C) and collected them 15 minutes, 24 hours or 48 hours later. For negative control, the cells were treated 15 minutes with 100% oxygen, transferred to the incubator with standard conditions and collected 15 minutes, 24 or 48 hours later. Western blotting analysis using aromatase- specific antibody demonstrated significant increase in aromatase protein expression in endothelial cells treated with desflurane and collected 48 hours later (Fig. 6 B).

## Discussion

In this study we could show that 1) aromatase inhibition using the pharmacological agent anastrozole attenuated desflurane-induced cardioprotection, 2) aromatase is expressed in the cardiovascular system of male mice, particularly in the endothelium and smooth muscle cells and 3) desflurane itself can induce aromatase expression in endothelial cells *in-vitro*.

To study the role of endogenous estrogens in anesthetic preconditioning in male mice, we performed pharmacological inhibition of aromatase using the highly selective aromatase inhibitor anastrozole prior to anesthetic preconditioning and subsequent ischemia/reperfusion experiments. We also investigated whether desflurane can modulate aromatase gene expression and studied its localization profile in the murine heart.

Desflurane is a potent cardioprotective volatile anesthetic which protective action in acute myocardial infarction model was shown by us and other groups [4], [20], [21]. Experimental data are supported by several clinical studies which showed less cardiac damage in patients receiving the volatile anesthetic desflurane compared to control patients [22]–[24]. Another study compared the effects of sevoflurane versus total intravenous anesthesia, in terms of postoperative cardiac troponin I release in patients undergoing noncardiac surgery. In this study patients undergoing noncardiac surgery did not benefit from anesthesia based on halogenated anesthetics [25]. In another study a longitudinal study of 34,310 coronary artery bypass graft interventions performed in Italy estimated the risk-adjusted mortality ratio. The survey among 64 Italian centers showed that risk-adjusted mortality may be reduced by the use of volatile agents in patients undergoing coronary artery bypass graft surgery [26]. Another study compared volatile agents to total intravenous anesthesia on mortality in cardiac surgery. Indeed, volatile agents reduce myocardial infarction (NNT = 37) and mortality (NNT = 83) in cardiac surgery when compared to total intravenous anesthesia [27]. Although there currently is no definitive proof, it seems that volatile anesthetics have beneficial effects especially on mortality when compared with total intravenous anesthesia.

Our study confirmed that even short- term application of desflurane was sufficient to reduce efficiently cardiac damage in acute myocardial infarction model. Volatile anesthetic- mediated cardioprotection was efficient in male patients and in experimental animal studies but only several studies analyzed gender- specific differences in anesthetic preconditioning. Wang et al. observed female- gender specific reduction in infarct size and showed that isoflurane preconditioning did not produce additional cardioprotection [9]. Similarly, Kitano et al. studied effects of isoflurane preconditioning of male and female mice which underwent reversible middle cerebral artery occlusion. Isoflurane- preconditioned young and middle- aged male mice were neuroprotected whereas female mice were not affected by isoflurane preconditioning [28]. The protective role of locally synthesised female sex hormones estrogens in men was analyzed by Lew et al. where it was demonstrated that local estrogen synthesis is required for normal endothelial function whereas inhibition of estrogen producing enzyme aromatase resulted in impairment of flow-mediated dilation in young healthy men [17]. Our observation that aromatase was among the genes which were upregulated by desflurane in the heart and it was abundantly expressed in endothelial and in smooth muscle cells in male mouse heart, let us hypothesize that aromatase might also be involved in desflurane- induced cardioprotection in cardiac ischemia/reperfusion model. For our study, we used a highly selective fourth generation aromatase inhibitor anastrozole [29]. We demonstrated that aromatase inhibition by anastrozole at therapeutic concentrations (1 mg/kg BW) efficiently reduced local estrogen synthesis and thus blunted desflurane- induced cardioprotection in male mice without influencing cardiac function.

Estrogen acts not only via its cognate receptors (genomic action), but can also directly influence nitric oxide generation and bioavailability (non- genomic action). Estrogens were shown to influence endothelial NO synthase (eNOS) activity and expression in rat aortic endothelial cells leading to increased NO generation and improvement of endothelial function in spontaneously hypertensive female rats [30]. In acute myocardial infarction in dogs, acute estrogen application resulted in reduced myocardial infarct size in both, male and female dogs whereas application of a NOS inhibitor abolished estrogen mediated cardioprotection [31]. Therefore, it is plausible to speculate that downstream effects of aromatase inhibition comprise ablation of estrogen synthesis leading to reduce NO production and/or stability and, subsequently, impairment of desflurane mediated cardioprotection in ischemia/reperfusion model.

Taken together, our data indicate that anastrozole is a potent aromatase inhibitor under *in vivo* conditions and is applicable for pharmacological studies of aromatase function. Also, anastrozole effectively blocked volatile anesthetic desflurane-induced preconditioning against myocardial infarction. Therefore, we conclude that estrogens at least partially contribute to desflurane-mediated cardioprotection in male mice. It is tempting to speculate that estrogen- like substances, such as estrogen receptor isoform- specific ligands described previously [30], [32], [33], might help to reduce cardiac damage following acute myocardial infarction in men. Thus, our findings may have important clinical implications and future clinical studies should identify whether sex could be a predictor of cardiac protection with volatile agents.

### Study Limitations

Due to application of Evans blue to assess myocardial infarction size, we could not measure estrogen and cyclic guanosine monophosphate levels (cGMP, a surrogate parameter for NO generation) in the plasma.
